# Robotic Materials Transformable Between Elasticity and Plasticity

**DOI:** 10.1002/advs.202206637

**Published:** 2023-02-15

**Authors:** Xinyuan Wang, Zhiqiang Meng, Chang Qing Chen

**Affiliations:** ^1^ Department of Engineering Mechanics CNMM and AML Tsinghua University Beijing 100084 P. R. China

**Keywords:** elasticity‐plasticity transformation, neutral stability, robotic material, shape sensing

## Abstract

Robotic materials, with coupled sensing, actuation, computation, and communication, have attracted increasing attention because they are able to not only tune their conventional passive mechanical property via geometrical transformation or material phase change but also become adaptive and even intelligent to suit varying environments. However, the mechanical behavior of most robotic materials is either reversible (elastic) or irreversible (plastic), but not transformable between them. Here, a robotic material whose behavior is transformable between elastic and plastic is developed, based upon an extended neutrally stable tensegrity structure. The transformation does not depend on conventional phase transition and is fast. By integrating with sensors, the elasticity‐plasticity transformable (EPT) material is able to self‐sense deformation and decides whether to undergo transformation or not. This work expands the capability of the mechanical property modulation of robotic materials.

## Graphical abstract

A robotic material that can transform between elasticity and plasticity is developed. The transformation does not depend on phase transition and is fast. By integrating with sensors, the elasticity‐plasticity transformable material is capable of deformation self‐sensing and deciding whether to transform or not. Thereby, it demonstrates a prototype of robotic materials with blurred boundary between materials and robots.

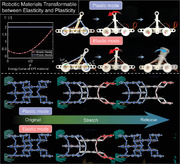

## 1 Introduction

Mechanical metamaterials are artificial materials with periodic structures.^[^ ^1^, ^2^ ^]^ Varying the microstructure of metamaterials can make it possible to achieve unique and even exotic mechanical properties, such as negative Poisson's ratio,^[^ ^3^, ^4^ ^]^ ultra‐high stiffness,^[^ ^5^, ^6^ ^]^ and multi‐stability,^[^ ^7^, ^8^ ^]^ that conventional materials usually do not possess. Despite their rich properties, mechanical metamaterials once fabricated, their in‐service properties cannot be easily changed to adapt to varying environments. Recently, there is increasing interest in metamaterials with tunable mechanical properties, which can be achieved, for instance, by creating new contacts,^[^ ^9^, ^10^ ^]^ structural buckling,^[^ ^11^ ^]^ or hinge rotation.^[^ ^12^, ^13^ ^]^ Among various available construction methods,^[^ ^11^, ^14^, ^15^, ^16^, ^17^, ^18^, ^19^, ^20^ ^]^ tensegrity structures^[^ ^21^ ^]^ are promising for the modulation of mechanical properties because it is easy to embed active elements into their constituent rods and tensile springs. It has also been shown that such structures can exhibit zero stiffness for deliberately designed geometries.^[^ ^21^, ^22^, ^23^ ^]^ Under the circumstances, the potential energy is constant and the structures are neutrally stable, giving rise to enhanced deformability.^[^ ^24^ ^]^

Robotic materials – that combine actuation, sensing, computation, and communication – become an emerging topic.^[^ ^25^, ^26^, ^27^, ^28^, ^29^ ^]^ With advances in actuation and sensing technologies,^[^ ^30^ ^]^ building programmable matter is now possible (e.g.,^[^ ^31^, ^32^ ^]^). They are expected to have autonomous computation capabilities to process information.^[^ ^33^, ^34^, ^35^, ^36^ ^]^ To this end, a number of studies have been reported, e.g., mechanical logic computation^[^ ^35^, ^37^, ^38^ ^]^ and physical system‐based neural networks.^[^ ^39^, ^40^ ^]^ After acquiring information and performing computation, robotic materials can tune their mechanical response to suit the environment. This capability is often achieved by incorporating special controllable active elements into a metamaterial, such as thermally driven artificial muscles,^[^ ^41^ ^]^ liquid crystal elastomers,^[^ ^42^, ^43^ ^]^ pneumatic actuators,^[^ ^44^, ^45^ ^]^ shape‐memory materials,^[^ ^46^ ^]^ and magnetic materials.^[^ ^47^ ^]^ Robotic materials that can actively adjust stiffness^[^ ^48^ ^]^ and Poisson's ratio^[^ ^49^ ^]^ have been developed so far. However, most available studies focus on regulating the property of materials that is either elastic or plastic, but not transformable between them.

In this paper, a tensegrity structure‐based robotic material is developed so that it can transform between an elastic solid (i.e., its deformation is recoverable upon removing loading) and a plastic solid (i.e., its deformation can only be partly recoverable upon unloading), giving rise to elasticity‐plasticity transformation. The material is also designed to have the ability to sense its own deformation with sensors and can automatically convert between elasticity and plasticity based on its deformation upon loading, demonstrating a possible pattern of robotic materials.

## 2 Results

**Figure 1:**
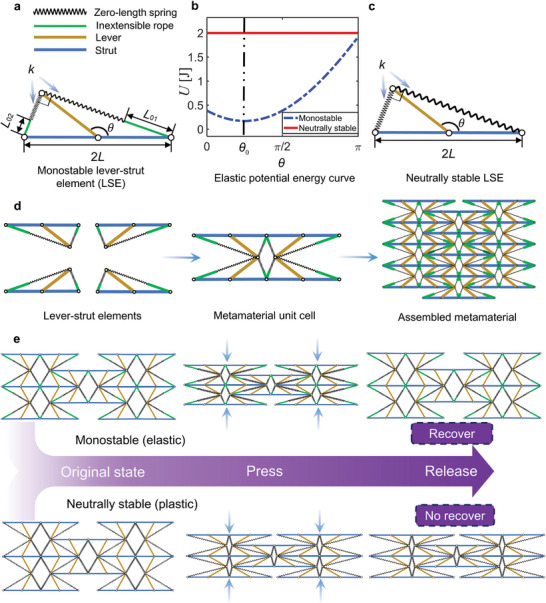
The basic principle of EPT materials. a) The structure of LSE in elastic mode. b) The elastic potential energy curves of LSE in the elastic (monostable) and plastic mode (neutrally stable). c) The structure of LSE in plastic mode. d) The tessellation procedure from LSE to a metamaterial. e) Different responses of EPT material's elastic and plastic mode under a compressive load. The rebound of the EPT material occurs after load release in the elastic mode, while it does not occur in the plastic mode.

The basic constituent element of the elasticity‐plasticity transformable (EPT) robotic metamaterial is illustrated in **Figure** **1**a, including a lever (shown as a brown line), a strut (blue), and two springs. Generally, the length of a deformed spring can be considered as the sum of its original length and its elongation, equivalent to an inextensible rope (green line) and a zero original length spring (zigzag line). One end of the lever is pin‐jointed at the center of the strut, allowing relative rotation between these two parts. The other end of the lever is connected to the two ends of the strut via two springs of the same stiffness *k*. The lengths of the lever and the strut are *L* and 2*L*, respectively, with the initial lengths of the springs being *L*_01_ and *L*_02_. The angle between the lever and the right half of the strut is defined as *θ*. In the following, this basic element is referred to as a lever‐strut element (LSE).

It should be pointed out that the stiffness of LSE can be finite or zero, depending upon its geometrical parameters. The potential energy of LSE has the form of

(1) $$U=\frac{1}{2}k\left[4L^{2}+L_{0}^{2}-4LL_{0}\cos\left(\frac{\theta -\theta_{0}}{2}\right)\right]$$

in which $\theta_{0}=2\arctan\left(L_{01}/L_{02}\right)$ and $L_{0}=\sqrt{L_{01}^{2}+L_{02}^{2}}$. From Equation (1), the elastic recovery moment *M*_*e*_ associated with *θ* can be obtained as

(2) $$M_{e}=\frac{dU}{d\theta }=kLL_{0}\sin\left(\frac{\theta -\theta_{0}}{2}\right)$$

which shows that *L*_0_ dictates the stiffness, and the ratio between *L*_01_ and *L*_02_ (i.e., *θ*_0_) determines the equilibrium position. First, consider the case *L*_0_ ≠ 0. The elastic potential energy curve of a typical LSE with *θ*_0_=*π*/4, *L*_0_ = 1, and *k* = 1 is plotted in Figure 1b as the dashed blue line, showing mono‐stability. When the structure deviates from its equilibrium state at *θ* = *θ*_0_, the elastic recovery force drives it back to *θ*_0_. When the initial length of the constituent springs of the LSE is zero (i.e., *L*_0_ = 0 in Figure 1c), one can see from Equation (1) that the potential energy becomes constant (*U* = 2*kL* ^2^) and is independent of *θ*, see the red solid line in Figure 1b. Consequently, the elastic recovery force is zero, according to Equation (2). Therefore, an LSE with zero initial‐length springs can be in equilibrium for any *θ*, which is referred to as “neutrally stable”^[^ ^24^ ^]^ or zero stiffness. Due to friction, a neutrally stable LSE can have an infinite number of equilibrium positions, allowing the structure to generate complex deformation patterns without the need of overcoming elastic potential energy.

It should be pointed out that the concept of zero stiffness in EPT materials is different from the quasi‐zero stiffness in vibration isolation structures. For the latter, a zero stiffness can only exist at particular positions, whereas the neutral stability in EPT materials, in theory, allows the materials to have zero elastic force at any position. The Young's modulus of existing stiffness programmable materials can vary hundreds of times.^[^ ^48^ ^]^ However, the lowest stiffness is not exactly equivalent to the zero stiffness in EPT materials. In contrast, EPT materials in a zero‐stiffness mode deform without the need of overcoming elastic potential energy, and can thus enhance their shape‐morphing capability. Note that in actual structures, friction is inevitable and can provide resistance to deformation for the structures in the zero‐stiffness mode, exhibiting plastic‐like properties and will not rebound after deformation. The zero‐stiffness mode is therefore referred to as “plastic mode”. For comparison, when elastic forces dominate, the structures will rebound after complete unloading, which is referred to as “elastic mode”.

**Figure 2:**
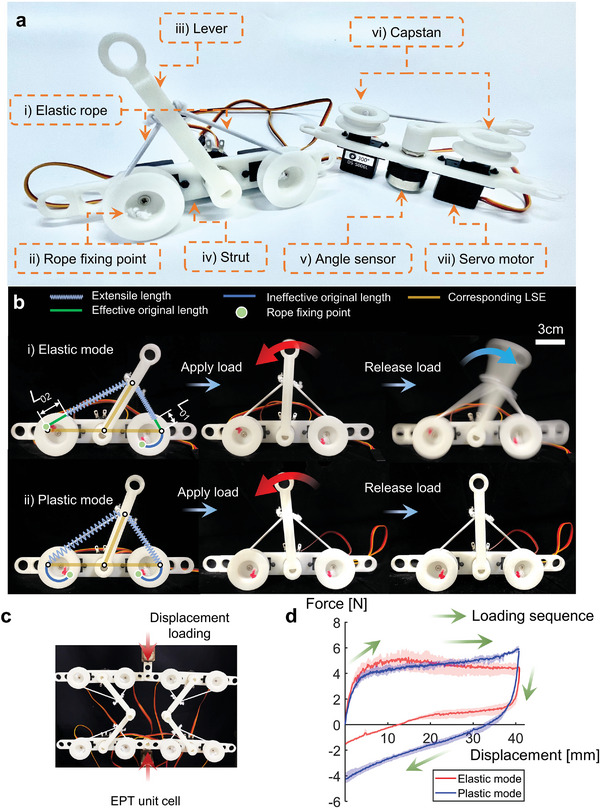
Implementation of the EPT unit. a) Main components of the EPT robotic material unit. b) The transformation between elastic and plastic modes. Unit behaves differently when the same load applies to it. The servo motor can control the effective original length of the elastic rope. c) The uniaxial cyclic loading test of the EPT unit cell. d) The measured force‐displacement curves of the EPT unit in elastic (red) and plastic (blue) modes.

With the LSE, a unit cell for constructing monostable and neutrally stable mechanical metamaterials can be designed, as illustrated in Figure 1d. The unit cell consists of four LSEs (Figure 1d). When the constituent LSEs are monostable, the deformation of the metamaterial is recoverable, showing elastic behavior (the top panel of Figure 1e). When the obtained metamaterial is made of neutrally stable LSEs, however, it exhibits plastic behavior, i.e., it can stay in its current configuration upon removal of loading (see the bottom panel of Figure 1e). Recall that whether an LSE is monostable or neutrally stable is dictated by the initial length of the springs. If the initial length of the springs is tunable from zero to a finite value, the resulting mechanical metamaterial is expected to be able to transform from plastic to elastic. To this end, we design the LSE with its *L*_01_ and *L*_02_ adjustable to enable the elasticity‐plasticity transformation of the metamaterial. Such an LSE with self‐regulation is referred to as an EPT element, composed of elastic ropes, levers, strut, angle sensor, capstans, and servo motors (**Figure** **2**a). The components colored in white are made of photosensitive resin by 3D printing. A potentiometer is used as the angle sensor and replaces the planer joint between the lever and strut to detect its rotation angle. The springs are now replaced by two elastic ropes. One end of the elastic rope is connected to the lever and the other end is fixed to the capstan. Servo motors are fixed on the strut and connected to capstans in order to control their rotation. The holes on the strut and lever are designed to connect with other units and allow them to rotate relatively to each other. The *L*_01_ and *L*_02_ of LSE can be adjusted by the rotation provided by the servo motors. (See Supporting information S4 for the installation method)

The EPT elements in elastic and plastic modes are shown in the leftmost part of Figure 2b. The corresponding structures are marked by brown lines. The green point shows the position of one end of the rope fixed on the capstan. The light blue zigzag lines denote the pure elongation part of the rope, equivalent to an elastic rope with zero original length. According to Equation (1), the original length of the elastic rope (i.e., the green line) determines the mechanical property of the LSE. In the elastic mode, *L*_01_ and *L*_02_ are equal to the lengths of the green lines (effective original length) in the top left of Figure 2b, respectively. The reason why the blue parts (ineffective original length) are not accounted for in *L*_01_ and *L*_02_ is that they correspond to inextensible ropes strung on the outside of the idealized models and do not change the spring elongation.

By controlling the rotation angle of the servo motors, the LSE can transform from elastic to plastic mode, and vice versa. If the length of the ineffective original length (blue) is equal to its complete original length, the length of the rest part of the elastic rope is due solely to the elongation of the rope. Under such a circumstance, the EPT element is equivalent to an LSE with zero initial length springs and is in plastic mode, as shown in the bottom left of Figure 2b. When the servo motors loosen the capstan and partly release the rope, the effective original length of the rope (green) is increased, giving rise to springs with a non‐zero original length. Accordingly, the EPT element is in elastic mode. The difference between the responses of the elastic and plastic mode can be seen from the right part of Figure 2b. After releasing the external load, the element in plastic mode will maintain its shape. On the contrary, it will recover to its original shape when in elastic mode. Therefore, controlling the servo motor's rotation can change the effective original length of the elastic rope, which in turn can control the switching of the element from plastic to elastic mode or vice versa (also see Supplemental Video S1).

By assembling four EPT elements as given in the middle of Figure 2c, an EPT unit cell is obtained. Compressive tests of the EPT unit cell under cyclic displacement‐controlled loading were conducted using a uniaxial testing machine (Zwick Z005). To facilitate the experiment, two pairs of struts connected laterally in the unit cell were fixed and not allowed to rotate relative to each other. The corresponding design parameters and mechanical models are shown in Materials S3, Supporting Information.

The measured force‐displacement curves for the elastic (red) and plastic (blue) modes are shown in Figure 2d, where the green arrows indicate the loading sequence. The dark curves in the experiment are the average results of three replicate experiments, and the shaded area represents their max fluctuation range. During forwarding loading, the axial force of the EPT cell is always opposite to the direction of loading in both modes. In the return trip of the elastic mode (unloading), its axial force does positive work. The intersection of the curve with the transverse axis is at 13 mm, so it can return to its initial state after releasing loads. On the other hand, the axial force of the unit in the plastic mode is still opposed to the loading direction in the return trip and thus exhibits plastic behavior. The curve's intersection with the transverse axis is at a displacement equal to 35 mm. If the unit is released at the point of maximum stroke, it cannot return to its original shape.

**Figure 3:**
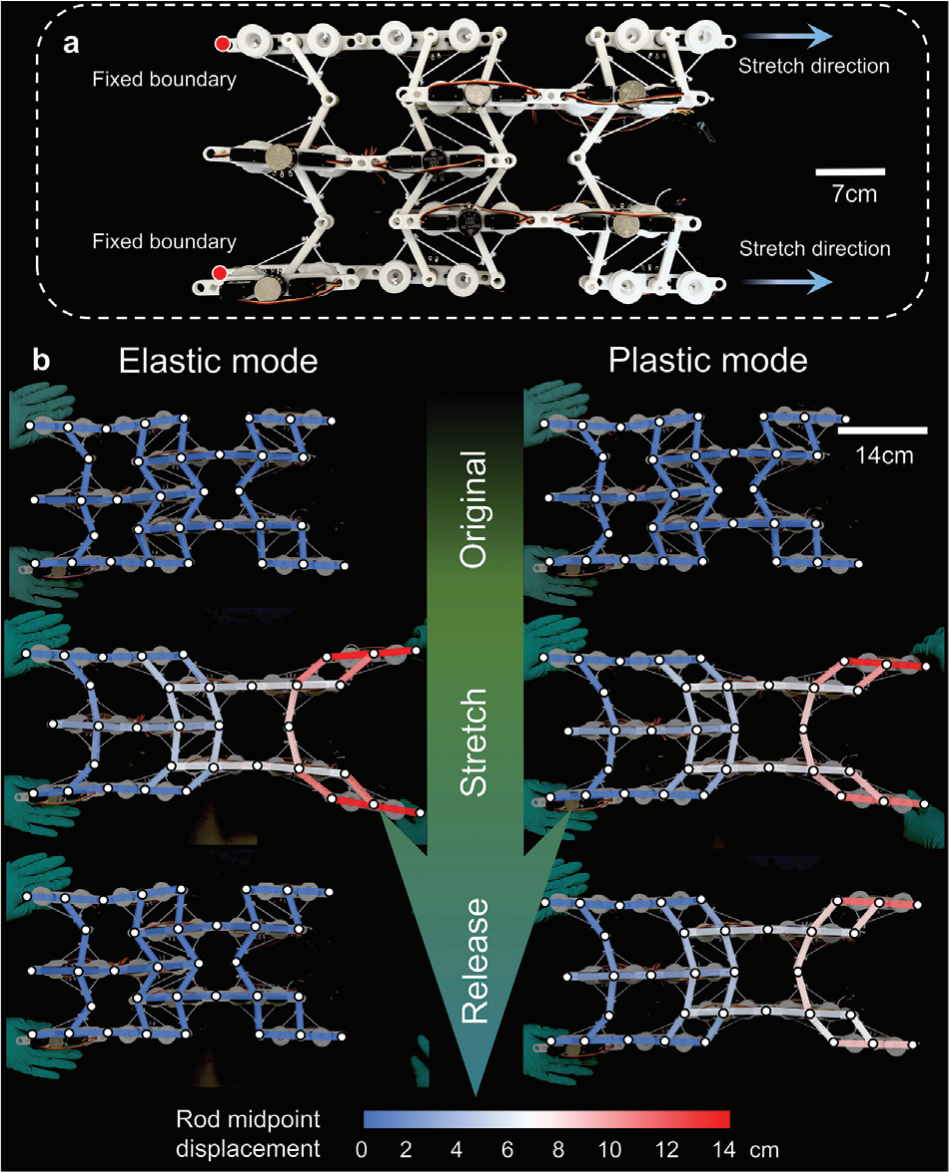
Demonstration of the EPT metamaterial's different modes. a) Assembled EPT metamaterial. b) Tensile responses of its elastic and plastic modes.

The EPT unit cell can be tessellated to form EPT metamaterials, with a typical example shown in **Figure** **3**a (see Supporting Information S4 for the assembly method). This metamaterial consists of three complete EPT cells and two half EPT cells (upper and lower right corners) controlled by 36 servo motors, allowing for elasticity‐plasticity transformation. All motors are operated through a microcontroller (Hiwonder LSC‐24‐V2.3). As shown in Figure 3a, in the stretching experiment, the red dot on the left side is fixed, and the arrow on the right side indicates the direction of stretching. The measured deformations of the metamaterial under stretching in the horizontal direction are shown in Figure 3b, with the elastic and plastic modes given in the left and right columns, respectively. The rods are marked with different colors to indicate their midpoints’ horizontal displacement obtained by the digital image correlation (DIC) method. In the elastic mode, the EPT metamaterial is seen to recover its original shape after completely releasing the stretching. In the plastic mode, however, the metamaterial retains most of its deformed shape after the stretching loading is removed. The record of this experiment is shown in Video S2, Supporting Information. As pointed out earlier, the transformation from plastic to elastic mode of the EPT mechanical metamaterial and vice versa can be easily achieved by adjusting the rope length controlled by the servo motors. This is much faster compared to the elasticity‐plasticity transformation based on the heating‐triggered phase transition in a previous study.^[^ ^24^ ^]^ The easy‐to‐implement, fast elasticity‐plasticity transformation is beneficial for the adaptation of the proposed mechanical metamaterial to a rapidly changing environment.

**Figure 4:**
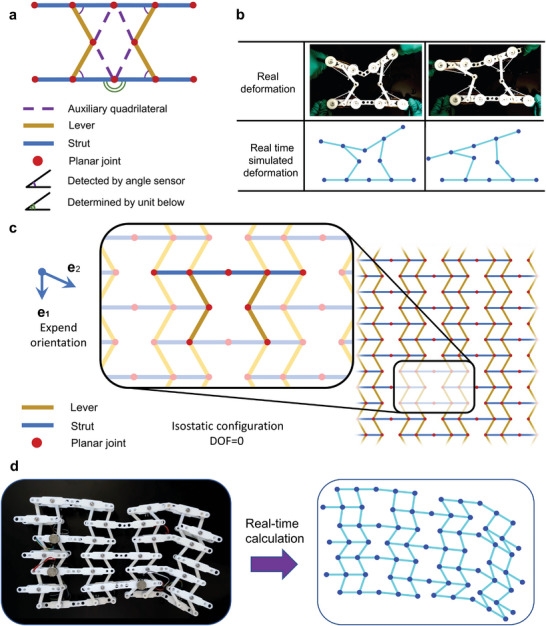
Deformation sensing of EPT robotic material. a) One EPT unit has five DOFs. Angle sensors provide the value of four angles (purple) and the neighbor unit provides the other one (green). In this way, the deformation of the EPT unit can be determined. b) By connecting robotic material with a PC, the deformation of the EPT unit can be simulated in real‐time. c) The average DOF of EPT unit is zero when it expands infinitely, making the structure an isostatic pattern. This characteristic means one can calculate the deformation of all units through the deformation of the boundary. d) Deformation sensible metamaterial. Due to the monostable nature of the EPT material, only 11 sensors are needed to determine the shape of the structure in this metamaterial consisting of 8 cells.

The proposed EPT mechanical metamaterial can not only enable transformation between elastic and plastic modes but also sense its shape morphing by analyzing the rotation angles of the constituent elements. As marked in **Figure** **4**a, an EPT unit cell consists of four struts, four levers, and eight planar hinges and, according to Maxwell's theory,^[^ ^50^ ^]^ has five degrees of freedom (DOFs). For each EPT unit cell, there are four sensors to measure the angles between the lever and the strut, i.e., the denoted inclined purple angles in Figure 4a. Without losing generality, the green angle in Figure 4a can be assumed to be known, because it can be inferred from the neighboring cells. Since the four purple angles can be obtained from the sensors, the lengths of the four sides of the auxiliary quadrilateral, represented by the purple dashed lines in Figure 4a, can be determined as well. Moreover, since the green angles are known, the angles of this quadrilateral can also be evaluated. Therefore, with the help of the angle sensors, real‐time shape tracking of the unit cell can be achieved as follows. First, the angle information of the EPT unit cell is read from sensors by an Arduino mega 2560 microcontroller and transferred to a computer via serial communication. Then, the deformed shape of the EPT unit cell can be determined (for the algorithm see Materials S1 and S2, Supporting Information), as given in Figure 4b (also see Video S3, Supporting Information).

When the EPT unit is tessellated to form a metamaterial, one might predict that a total of 4*n* ^2^ sensors would be required for *n* × *n* unit cells. However, the number of sensors required in a metamaterial is much less than the above prediction. Figure 4c shows a unit cell in the interior, not on the boundary, of an EPT metamaterial. Applying Maxwell's law to this cell, the number of DOFs is found to be zero, which can be generalized to other interior unit cells. The case of a periodic structure in which the internal unit has zero degrees of freedom is referred to as isostatic.^[^ ^51^ ^]^ In such cases, all the DOFs of the metamaterial lie on the boundary. The number of sensors required to determine the shape of an EPT metamaterial of *m* × *n* can be obtained as

(3) $$N=\left\{\begin{array}{c} 2n+2m-1,m\;\text{is odd}\\ 2n+2m-2,m\;\text{is even} \end{array}\right.$$

which is much less than 4*mn* when *n* or *m* is large (see Material S2, Supporting Information). Therefore, once the boundary shape is determined, the interior shape of the metamaterial can be calculated through the constraint equation. This property of the EPT material unit reduces the number of sensors required for shape sensing and decreases the complexity of a control system.

Figure 4d shows the self‐sensed shape of an EPT metamaterial with 3 × 3 arrays of unit cells. Based on the analysis above, the deformed shape can be fully determined with only 11 sensors on the boundary instead of 36 sensors for the entire material. The left panel shows the actual shape of the EPT metamaterial, while the right panel shows the computed shape based on the angle information from the sensors (See Material S2, Supporting Information, for the computation algorithm and method to increase accuracy). One can see from Figure 4d that the self‐sensed (calculated) shape is in good agreement with the actual deformation. This example also validates an important feature of the proposed EPT metamaterial, i.e., the information on its global deformation can be solely determined from that of the boundary cells. This feature dramatically reduces the number of required sensors, thus achieving a balance between deformability and the number of sensors (see Video S3, Supporting Information).

**Figure 5:**
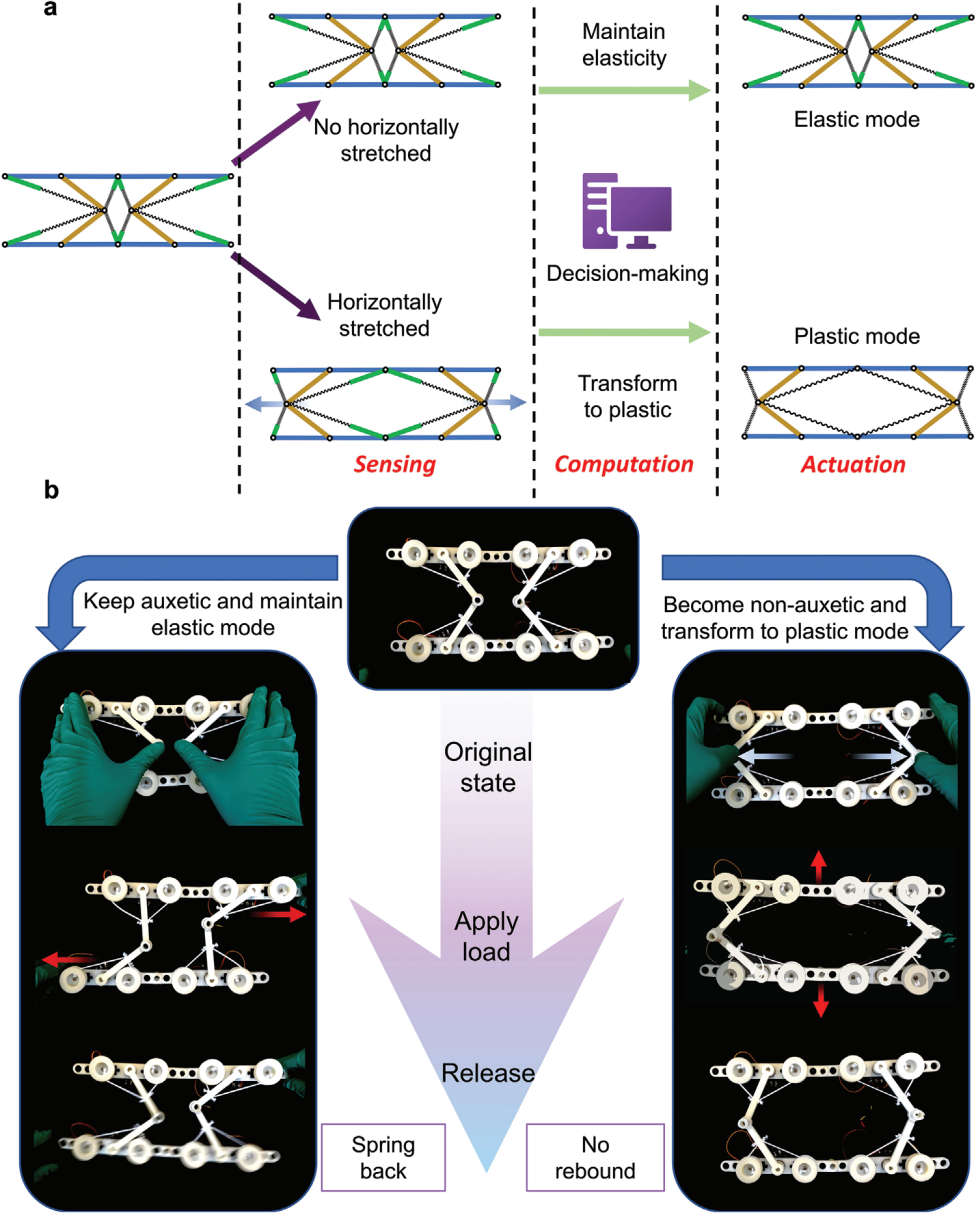
Deform‐dependent mechanical property control of EPT robotic material. a) The diagram of deform‐dependent mechanical property control. b) Experimental demonstration of autonomous tuning of the mechanical property of the EPT robotic material. When in auxetic pattern, the unit springs back after load releasing while in non‐auxetic pattern, the unit can keep equilibrium at any position.

With the capability of elasticity‐plasticity transformation and deformation self‐sensing, the EPT metamaterial can achieve deformation‐dependent mechanical property regulation, as shown in **Figure** **5**a. By way of example, the EPT material is designed to show different mechanical responses of auxetic or non‐auxetic. Figure 5b and Video S4, Supporting Information, experimentally demonstrate the idea discussed above. The unit cell can keep auxetic or be stretched to a non‐auxetic configuration. In the auxetic configuration, it can return to its initial state after the load is released. When the cell is stretched laterally into a non‐auxetic configuration, it automatically enters the plastic mode. Thereby, the EPT cell can maintain its deformed shape after releasing the load. In fact, by designing algorithms that accurately identify external loads, robotic materials are expected to be able to better tailor their properties and thus have the capability of “material properties upon demand”.

## 3 Discussion

A robotic material (i.e., EPT material) with controllable transformation between elasticity and plasticity is developed. The transformation is achieved by tuning the geometry of the constituent elements instead of material phase transition and can thus enable rapid transition. When in plastic mode, the EPT material can dissipate energy and is expected to be used for impact attenuation, among others. Compared to conventional plastic materials, the EPT material can actively recover its original shape by converting to its elastic mode after impact. It should be noted that the size of the EPT unit in this study is on the order of centimeters and can be further reduced by using miniature actuators such as thermally driven artificial muscles,^[^ ^41^, ^42^ ^]^ liquid crystal elastomers,^[^ ^43^ ^]^ or shape memory polymer.^[^ ^46^ ^]^

The isostatic characteristic of the EPT material facilitates the design of robotic materials to have the deformation self‐sensing capability with only a few sensors needed, which is in turn employed to achieve the deformation‐dependent regulation of the transformation between elasticity and plasticity. This study adds new options for robotic materials that can modulate mechanical response. In the future, by combining mechanics and information, the boundary between materials and robots is expected to be blurred.

## Conflict of Interest

The authors declare no conflict of interest.

## Supporting information

Supporting Information

Supplemental Video 1

Supplemental Video 2

Supplemental Video 3

Supplemental Video 4

## Data Availability

The data that support the findings of this study are available in the Supporting Information of this article.
