# Supplementary material for: Robotic Materials Transformable Between Elasticity and Plasticity
Source: Adv Sci (Weinh). 2023 Feb 15;10(13):2206637. doi: 10.1002/advs.202206637 (PMC10161124; doi:10.1002/advs.202206637)
Supplement: Supplementary file 1 — Supporting Information [file ADVS-10-2206637-s001.pdf]

Supplementary Material for

Robotic Materials Transformable between Elasticity and Plasticity

Xinyuan Wang, Zhiqiang Meng, *and Chang Qing Chen *

Department of Engineering Mechanics, CNMM and AML, Tsinghua University

Beijing 100084, P.R. China

S1. Geometry and Shape sensing of the EPT cell

To extract the deformation information of the EPT material, it is necessary to discuss its degrees of freedom (DOFs). According to Maxwell's theory, the DOF of a mechanism is given by:

$$\text{DOF}=3(n-1)-3g+\sum_{i=1}^{g} f_i \tag{S1}$$

in which n stands for the number of rigid bodies, g is the number of joints, and f_i refers to the DOF of each joint. A single EPT unit (shown in Figure S1) consists of eight rods ($n=8$) and eight planer joints (i.e., $g=8$, $f_i=1$ for $i=1,...,8$). Accordingly, its number of DOFs is five. Each EPT unit is equipped with four angle detectors marked by purple. We assume the green angle $\angle\text{EAF}$ in Figure S1 can be determined by its neighboring unit cell. Therefore, the shape of the EPT unit can be determined by these five angles. Since the shapes of the four isosceles triangles are known, the quadrilateral ABCD marked in purple can be solved to obtain the shape of the EPT cell. In $\triangle\text{ABF}$, $\text{AF}=\text{BF}=L$ and AB can thus be obtained by the law of sine.

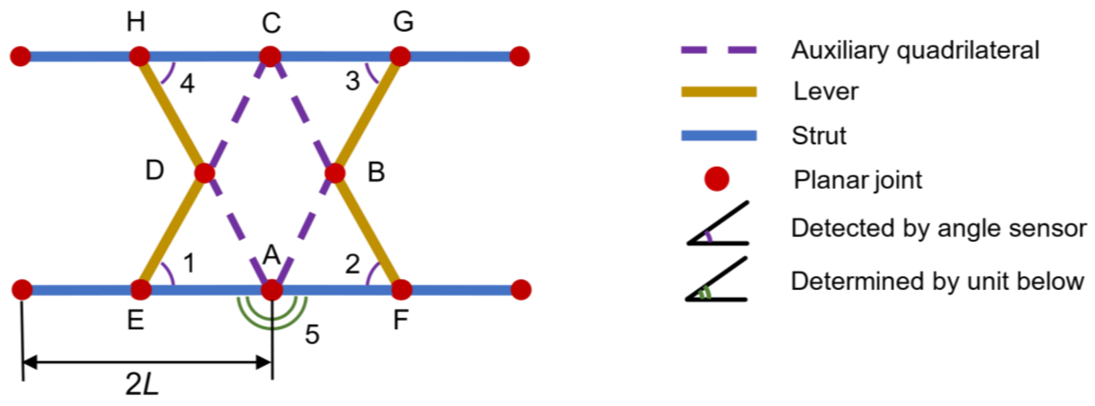

Figure S1. The geometry of EPT unit cell.

The other sides of the quadrilateral $ABCD$ can be obtained in a similar way. With the four side lengths of the quadrilateral $ABCD$ determined, only one of the angles has yet to be determined. For instance, $\angle DAB$ can be obtained as:

$$\angle DAB = \angle EAF + \frac{1}{2}\angle 1 + \frac{1}{2}\angle 2 - \angle 5 \tag{S2}$$

Thus, BD can be calculated by the cosine theorem:

$$BD = \sqrt{AD^2 + AB^2 - 2AD \cdot AB \cdot \cos\angle DAB} \tag{S3}$$

The angle of the top of the quadrilateral can then be found as:

$$\angle DCB = \arccos\left(\frac{CD^2 + BC^2 - BD^2}{2CD \cdot BC}\right) \tag{S4}$$

Once the quadrilateral $ABCD$ has been completely determined, the other angles of the EPT cell can be solved similarly. By doing that, all angles of the EPT cell and the coordinate positions of each point can be determined.

S2. Deformation sensing of the EPT metamaterial

As shown in Figure 4c, the internal cell of EPT metamaterial has zero DOF, indicating that the deformation of this type of EPT cell can be fully determined by the deformation of its adjacent units. Unlike the deformation sensing of a single cell, an EPT cell in a metamaterial has many constraints, which reduce the degrees of freedom of the metamaterial. By installing angular sensors in the cells on the boundary, the

deformation of the entire metamaterial can be determined with a small number of sensors.

Due to a large number of constraints in the whole structure, it is necessary to rationalize the solution sequence to avoid simultaneously solving complex systems of equations. One possible solution procedure is shown in Figure S2. In the first step, the shape of the first column is solved from bottom to top; in the second step, the shape of each column is solved by repeating step 1 in the order from left to right.

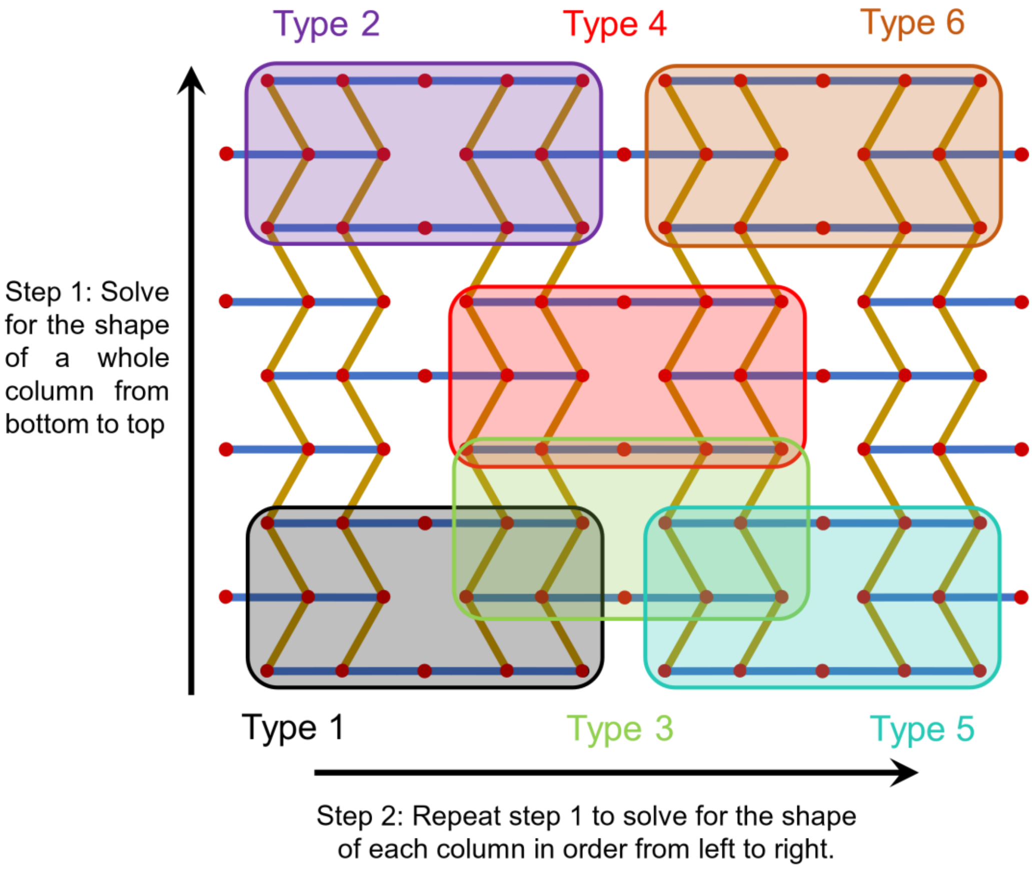

Figure S2. The shape calculation sequence and different types of constraint conditions in EPT metamaterial.

Following this sequence, there are six types of cells with different constraint conditions in the EPT metamaterial shown in Figure S2, and the corresponding known and unknown conditions are listed in Figure S3. The known angle information is from the angle detectors or neighboring cells whose shape have already been determined. Solving six different types of cell shapes can be generalized to two types of geometric

problems. One type is to find $\angle 3$, $\angle 5$ for known $\angle 1$, $\angle 2$, $\angle 4$; and the other is to find $\angle 2$, $\angle 3$ for known $\angle 1$, $\angle 4$, $\angle 5$. The six different positions of the cells require different numbers of sensors to determine their shape, and the angles that need to be detected are marked in purple in Figure S3.

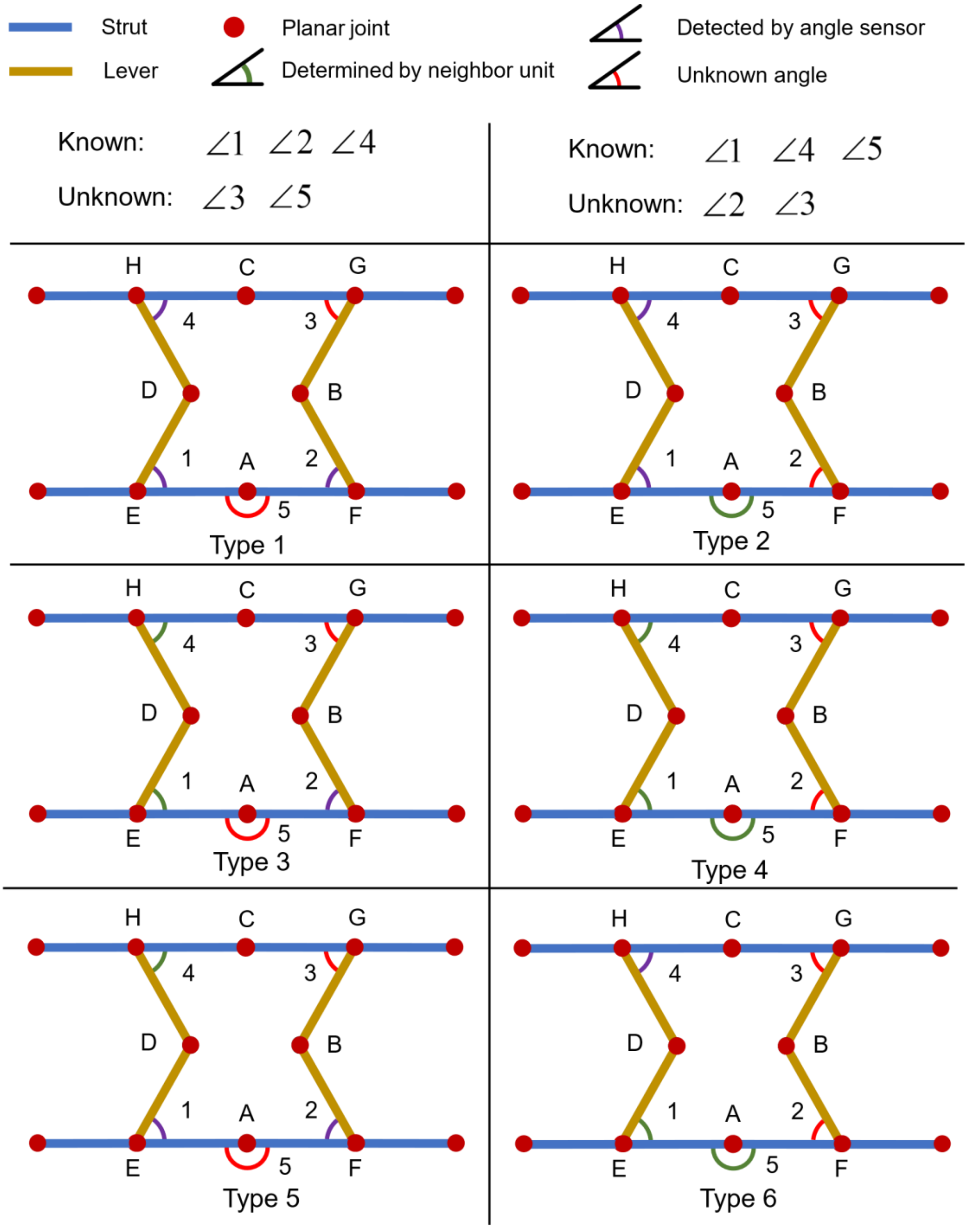

Figure S3. The conditions to solve the shape of the EPT unit in different types of cells.

Figure S4a shows the EPT configuration when the number of columns m is odd,

while b shows the case when m is even. Based on the analysis given in Figure S3, the number of sensors required to determine the shape of an EPT metamaterial of $m \times n$ can be obtained as

$$N = \begin{cases} 2n + 2m - 1, \, m \; is \; odd \\ 2n + 2m - 2, \, m \; is \; even \end{cases} \tag{S5}$$

This number is much smaller than $4mn$ as required by the method with each cell having one sensor. In the following, we introduces the metamaterial's shape-sensing algorithm used in this study and discusses how to solve the shape of the EPT cell under different constraints.

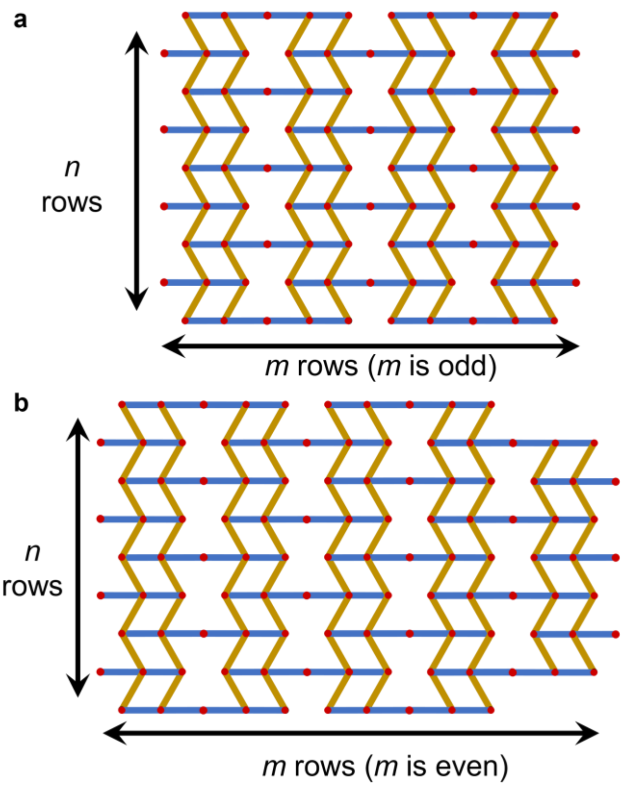

Figure S4. The calculation sequence and different types of constraint conditions in EPT metamaterial. a) The case when m is odd. b) The case when m is even.

S2.1 Solution procedure for cells -Type 1 & 3 &5

For the cells of type 1, 3, and 5, $\angle 1$, $\angle 2$, and $\angle 4$ are known, with $\angle 3$ and $\angle 5$ to be evaluated and $AE // CH$, $AF // CG$. Noting that $AE // CH$, one can get

$\mathrm{AC}=\sqrt{\mathrm{AD}^2+\mathrm{CD}^2-2\mathrm{AD}\cdot\mathrm{CD}\cos\angle\mathrm{ADC}}$, in which $\angle\mathrm{ADC}=\pi-\left(\angle 1+\angle 4\right)/2$. According to the parallel condition $\mathrm{AF}\,/\!/\,\mathrm{CG}$, $\angle 3$ can be obtained as

$$\angle 3=\arcsin\left(\frac{\mathrm{AC}^2-2L^2}{\sqrt{\left(\mathrm{AB}^2-2L^2\right)^2+4L^2\mathrm{AB}^2\cos^2\left(\angle 2/2\right)}}\right)-\arctan\left(\frac{\mathrm{AB}^2-2L^2}{2L\cdot\mathrm{AB}\cos\left(\angle 2/2\right)}\right) \quad \text{(S6)}$$

Therefore, $\mathrm{BC}=2L\sin\left(\angle 3/2\right)$, $\angle\mathrm{BAC}=\arccos\left[\left(\mathrm{AB}^2+\mathrm{AC}^2-\mathrm{BC}^2\right)/\left(2\mathrm{AB}\cdot\mathrm{AC}\right)\right]$, and the bottom angle can be solved. All conditions to solve the type 1, 3, and 5 cells are obtained.

S2.2 Solution procedure for cells -Type 2 & 4 & 6

For the cells of type 2, 4, and 6, $\angle 1$, $\angle 4$, $\angle 5$ are known, with $\angle 2$ and $\angle 3$ to be determined and $\mathrm{AE}\,/\!/\,\mathrm{CH}$ and $\mathrm{AF}\,/\!/\,\mathrm{CG}$. Figure S5 shows the geometry of a deformed EPT cell under the parallel constraints, i.e., $\mathrm{AE}\,/\!/\,\mathrm{CH}$, $\mathrm{CG}\,/\!/\,\mathrm{AF}$. Let the horizontal distance between A and C be a, the distance between AF and CG be h. The following relationships among a, h, $\angle 2$ and $\angle 3$ can be obtained

$$\begin{cases}\cos\angle 3-\cos\angle 2=\dfrac{a}{L}\\[2mm]\sin\angle 3+\sin\angle 2=\dfrac{h}{L}\end{cases} \quad \text{(S7)}$$

where a and h are known quantities. By solving Eq. (S7), $\angle 2$ and $\angle 3$ can be obtained as

$$\begin{cases}\angle 2=\arcsin\sqrt{\dfrac{a^2+h^2}{4L^2}}+\arctan\dfrac{a}{h}\\[2mm]\angle 3=\arcsin\sqrt{\dfrac{a^2+h^2}{4L^2}}-\arctan\dfrac{a}{h}\end{cases} \quad \text{(S8)}$$

The above derivation shows how one can solve for $\angle 2$ and $\angle 3$ under the condition that a and h are known. In these types of cells, $\angle 1$ and $\angle 4$ are known, using a principle similar to that of Eq. (S7), we can obtain

$$\begin{cases} a' = L(\cos\angle 1 - \cos\angle 4) \\ h' = L(\sin\angle 1 + \sin\angle 4) \end{cases} \tag{S9}$$

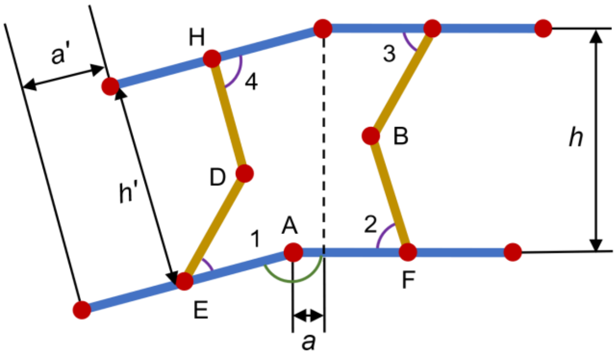

Figure S5. Geometry of EPT unit cell under parallel constraint.

The definition of a' and h' are shown in the left part of Figure S5. Since they are obtained under different reference systems, a' and h' derived from the above equation also need to be multiplied by a rotation matrix to obtain a and h. The angle of rotation is related to the bottom angle $\angle 5$.

$$\begin{pmatrix} a \\ h \end{pmatrix} = \begin{pmatrix} -\cos\angle 5 & -\sin\angle 5 \\ \sin\angle 5 & -\cos\angle 5 \end{pmatrix} \begin{pmatrix} a' \\ h' \end{pmatrix} \tag{S10}$$

where $\angle 5$ is marked as the green angle in Figure S5.

S2.3 Algorithm to improve shape-sensing accuracy

Using the above algorithm, calculations were performed in the order shown in Figure S2 for an EPT metamaterial, whereby 11 sensors are needed to fully determine its shape. The advantage of the algorithm is that it can be solved explicitly and thus can be calculated very fast. However, when the constraint of the structure is not idealized or one of the sensors fails, it may result in large errors. Therefore, a nonlinear programming-based algorithm is proposed here that can improve the shape sensing accuracy by increasing the number of sensors.

By way of example, the shape-sensing algorithm for a 3×3 EPT metamaterial is discussed. Figure S6 illustrates a possible sensor arrangement. When the procedure

shown in Figure S2 is adopted to solve for the unknown angles, the shape of the EPT material can be explicitly determined.

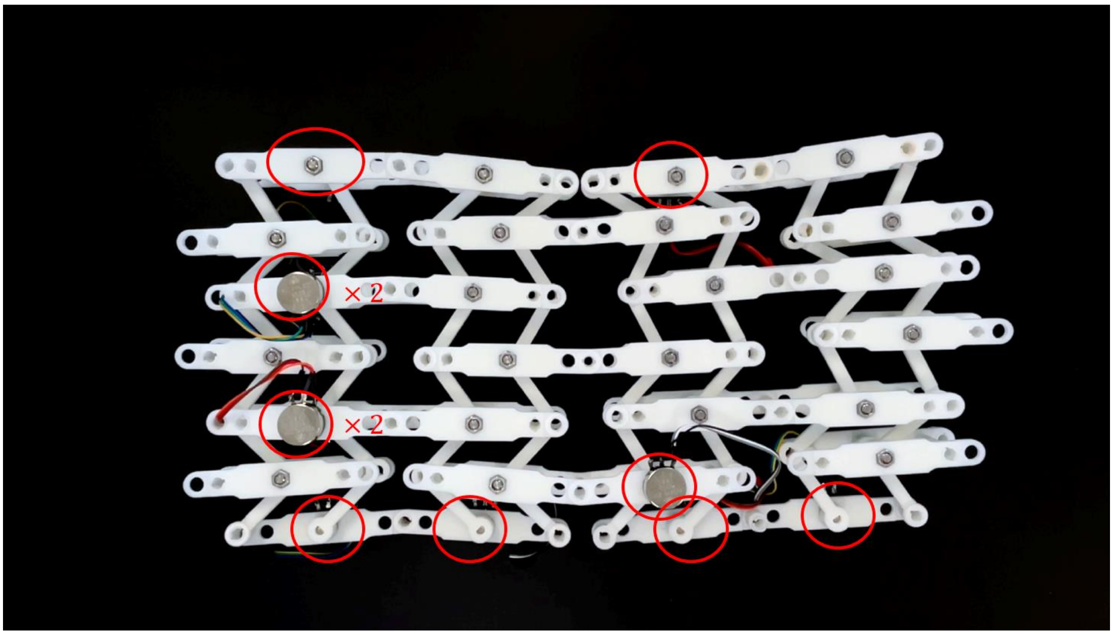

Figure S6. Sensors arrangement of shape sensing of an EPT metamaterial.

When the number of sensors is more than the number of degrees of freedom of the material, however, the constraint equation becomes overdetermined. Thereby, the solution of the shape can be transformed into a nonlinear programming problem, as formulated in the following. Define a nonlinear optimization problem, i.e.,

$$\begin{aligned} &\min\ f(\boldsymbol{\theta}) = \sum_{i=1}^{n} (F_i(\boldsymbol{\theta}) - \varphi_i)^2 \\ &s.t. \quad 0 \le \boldsymbol{\theta} \le \pi \end{aligned} \tag{S11}$$

where $\boldsymbol{\theta}$ is a set of vectors consisting of independent variables of the objective function, with the dimension equal to the degrees of freedom of the EPT material, φ_i is the sensors' reading, and $F_i(\boldsymbol{\theta})$ (calculated from $\boldsymbol{\theta}$) is the corresponding theoretical angle of φ_i. Taking a 3×3 EPT cell as an example, $\boldsymbol{\theta}$ of each element is marked by the red circles in Figure S6. It can also be another set of 11 independent angles, provided that it is convenient to solve $F_i(\boldsymbol{\theta})$. The dimension of φ_i depends

on the number of used sensors and can be equal to or more than 11. The sum of squares of the difference between φ_i (obtained from sensors) and $F_i(\boldsymbol{\theta})$ is taken as the objective function. A comparison of the shape-sensing results obtained using the optimization algorithm and that using the direct algorithm with only 11 sensors is shown in Figure S7. Compared to the direct algorithm, the optimization algorithm with 32 sensors shows a reduction of 75% in the mean square error of the actual shape. However, we want to emphasize that, although the optimization algorithm provides improved results, the number of required sensors increases significantly. As a result, the time of solution increases dramatically, which hinders real-time computing, sensing and actuation.

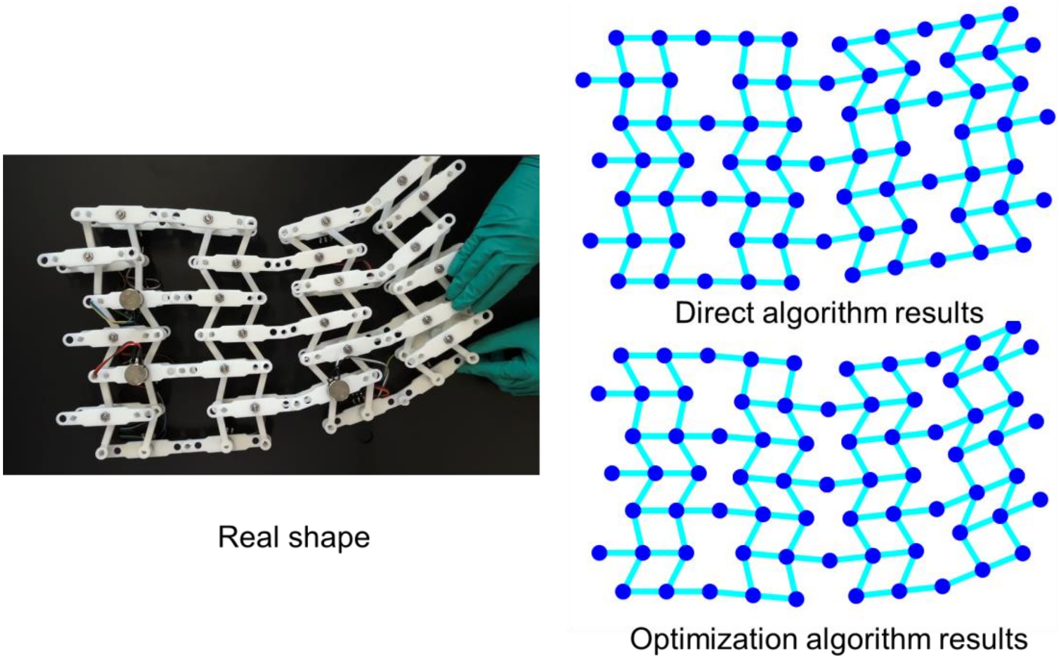

Figure S7. Comparison of the results obtained by the direct and optimization algorithm.

S3. Analysis of elasticity-plasticity transformation

The analysis of the LSE in the main text is limited to elastic deformation. In this section, frictional force is taken into account in characterizing the measured plastic behavior of the EPT material. The potential energy of LSE has the form of

$$U = \frac{1}{2}k\left[4L^2 + L_0^2 - 4LL_0\cos\left(\frac{\theta - \theta_0}{2}\right)\right] \tag{S11}$$

in which $\theta_0 = 2\arctan(L_{01}/L_{02})$ and $L_0 = \sqrt{L_{01}^2 + L_{02}^2}$. From Eq.(S11), the elastic recovery moment M_e associated with θ can be obtained as

$$M_e = \frac{dU}{d\theta} = kLL_0\sin\left(\frac{\theta - \theta_0}{2}\right) \tag{S12}$$

which shows that L_0 dictates the magnitude of the moment and the ratio between L_{01} and L_{02} determines the equilibrium position. In addition the elastic force, frictional force also contributes to the elasticity-plasticity transformation of the LSE, which allows the unit to be stable in plastic mode.

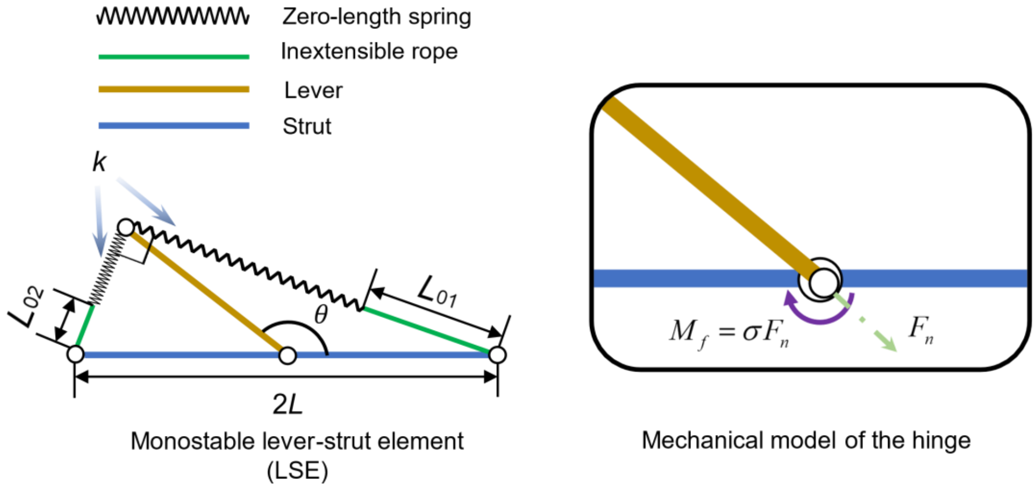

Figure S8. Model for the elasticity-plasticity transformation of the Lever-strut element.

As shown in Figure S8, the hinge can be assumed to have a linear frictional behavior. According to the force analysis, expression of the frictional resistance moment M_f can be obtained as

$$M_f = \sigma F_n \tag{S13}$$

in which F_n is the axial force of the lever, and σ is the frictional moment factor of the hinge. For simplicity, the following form of F_n is adopted:

$$F_n=\left[2L\sin\frac{\theta}{2}-L_{01}\right]\sin\frac{\theta}{2}+\left[2L\cos\frac{\theta}{2}-L_{02}\right]\cos\frac{\theta}{2} \tag{S14}$$

Eq. (S14) shows that the force at the hinge is generated only by the elasticity of the spring. The effect of the other loads can be characterized by varying σ. From Eq. (S14), the expression of frictional resistance moment can be obtained as

$$M_f=k\sigma\left[2L-L_0\cos\left(\frac{\theta-\theta_0}{2}\right)\right] \tag{S15}$$

To facilitate compression and tension tests, the two struts at top and bottom are fixed together when assembled as EPT cells, see Figure S7. The geometric relationship is as follows:

$$\theta=\arcsin\frac{y}{2L} \tag{S16}$$

Thus, according to the principle of virtual work, the force-displacement equation along the vertical direction of the EPT unit with friction taken into account can be obtained as:

$$F=\frac{kLL_0}{\sqrt{4L^2-y^2}}\sin\left(\frac{\theta-\theta_0}{2}\right)+\frac{k\sigma}{\sqrt{4L^2-y^2}}\left[2L-L_0\cos\left(\frac{\theta-\theta_0}{2}\right)\right]\mathrm{sign}(\mathrm{d}y) \tag{S17}$$

where $\mathrm{sign}(\mathrm{d}y)$ indicates that the direction of friction force is always opposite to the direction of displacement.

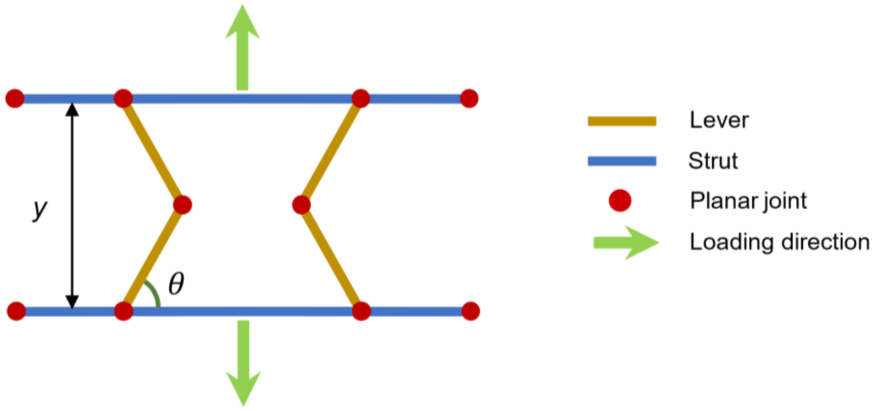

Figure S9. Schematic of the EPT cell used to perform the tensile test.

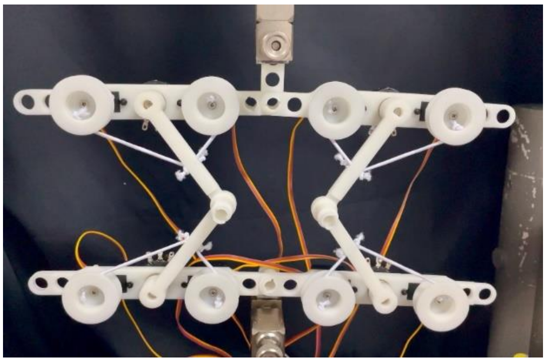

Figure S10. Axial cyclic test of EPT unit cell.

The experimental setup for the axial cyclic test of the EPT unit is shown in Figure S10. The force-displacement curves of the unit in the elastic and plastic mode were measured. The uniaxial testing machine is Zwick Z005. The tested unit cell had $L = 70\text{mm}$ and the initial height of the unit was 130mm. The cyclic loading was performed by first compressing and then stretching to the initial position. The maximum compression stroke was 40mm. The length of the elastic rope used in the EPT unit is 135mm with $EA = 4.146\text{N}$. The experimental results about the force-displacement curve are shown in Figure S11, with the length of the tested elastic rope being 88.6mm, and the loading rate of 20mm/min.

The force-displacement curves for the elastic and plastic modes under the cyclic loading test are shown in Figure S12 a and b, respectively. In the elastic mode, $L_0 = 55\text{mm}$; while in the plastic mode $L_0 = 30\text{mm}$. The loading rate is $30\text{mm} \cdot \text{min}^{-1}$. The red curves denote experimental measurement while the blue ones refer to the theoretical prediction by the model for the EPT unit. By fitting the two parameters k and σ against the experiment, we can get $k = 70\text{N} \cdot \text{m}^{-1}$ and $\sigma = 0.016\text{m}$. Although in the plastic mode, L_0 is not exactly equal to zero, the presence of friction can make it exhibit significant plastic characteristic and does not have enough elastic potential energy to restore it to its initial state.

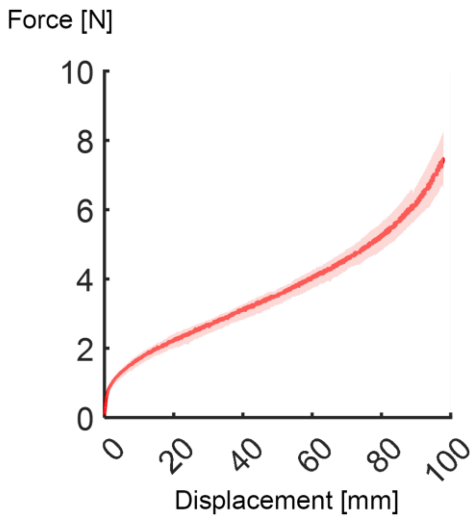

Figure S11. Force-displacement curve of elastic rope. The length of the elastic rope is 88.6mm.

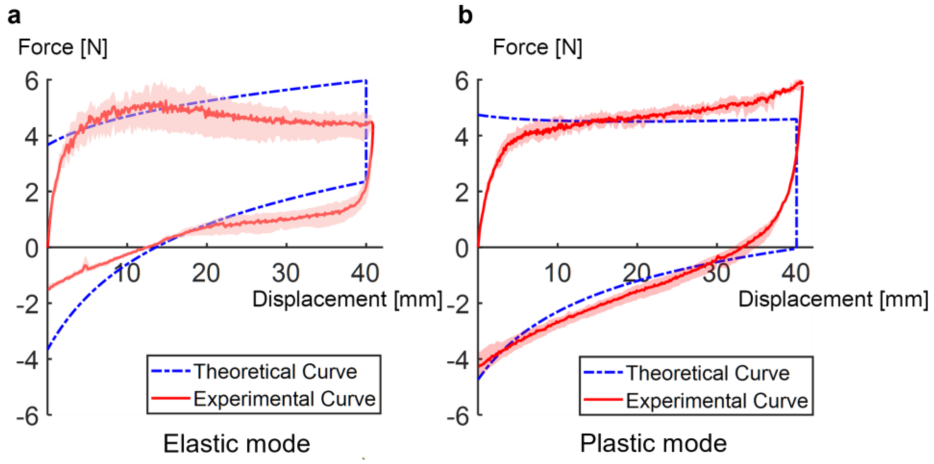

Figure S12. The force-displacement curves of EPT unit cell. a) Elastic mode. b) Plastic mode.

S4. Assembling of EPT robotic material

An EPT robotic material system is shown in Figure S13, consisting of a material part, a microcontroller, and a computer. The material part comprises actuators and sensors that receive instruction from the microcontroller to switch between elasticity and plasticity while transferring the deformation data to the microcontroller. The actuator for the EPT robot material is a servo motor (model DS-S006L, DFRobot) with a maximum rotation angle of $300°$ and a maximum torque of $11.7\mathrm{N\cdot cm}$.The angle

sensor uses a potentiometer (model RV24YN20S, Shenzhen Minheng Electronic Company) with a maximum resistance of $50\text{k}\Omega$.

The microcontroller model used here is Arduino Mega 2560. It receives signals from the material part of the robot through 11 analog signal ports. The microcontroller is connected to the computer through a USB port and transmits deformation data to the computer via serial communication. The computer writes the transferred data from the port to a text file in ASCII format via a Python script. This file is then read by MATLAB to reproduce the deformation of the EPT material in real-time.

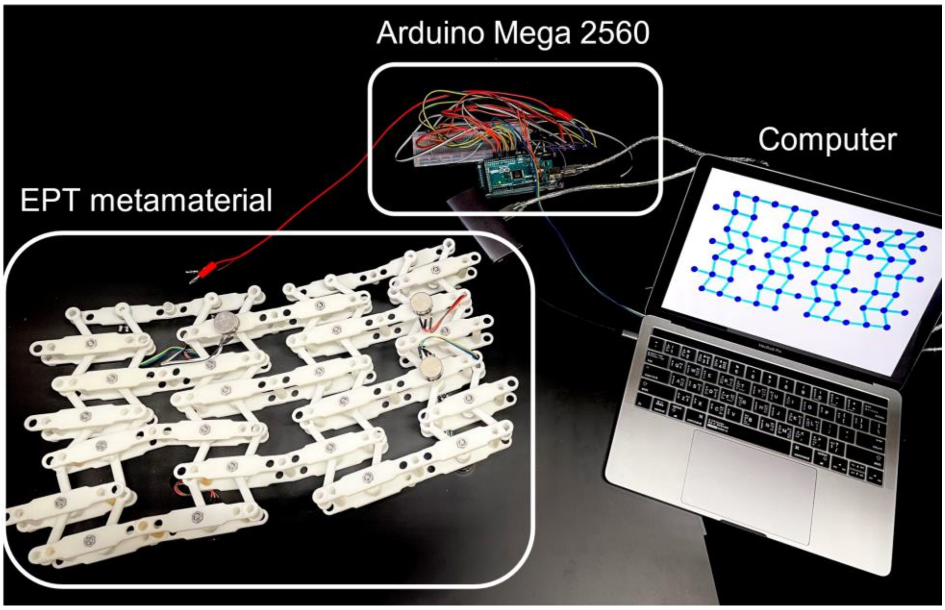

Figure S13. A typical EPT robotic material system.

The assembling of LSE and EPT unit cells are shown in Figure S14 and Figure S15, respectively. By tessellating the EPT unit cells, an EPT robotic material can be obtained, see Figure S16. The arrows indicate the installation direction.

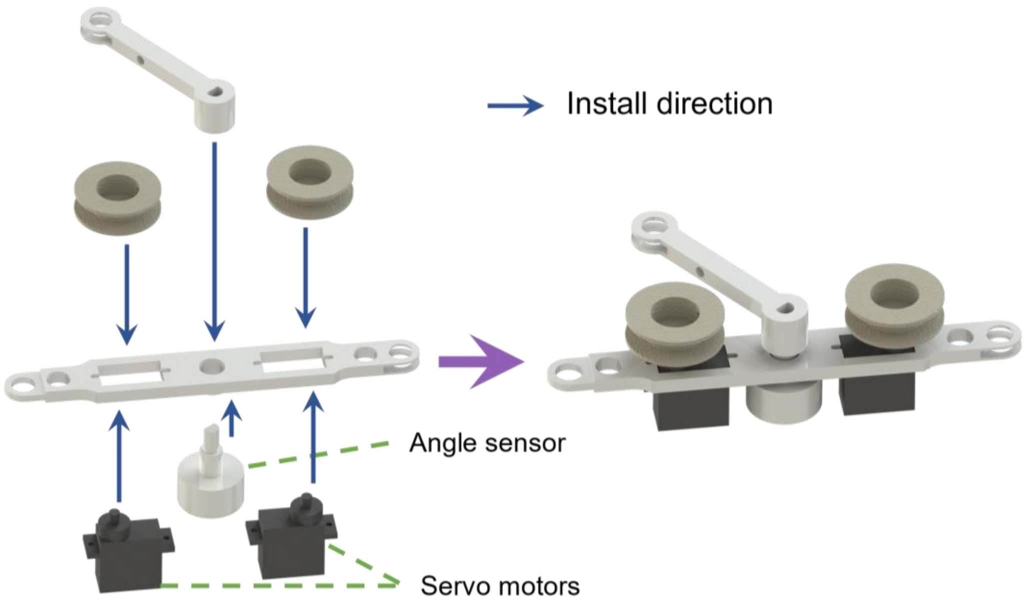

Figure S14. Procedure for assembling LSE and its key components.

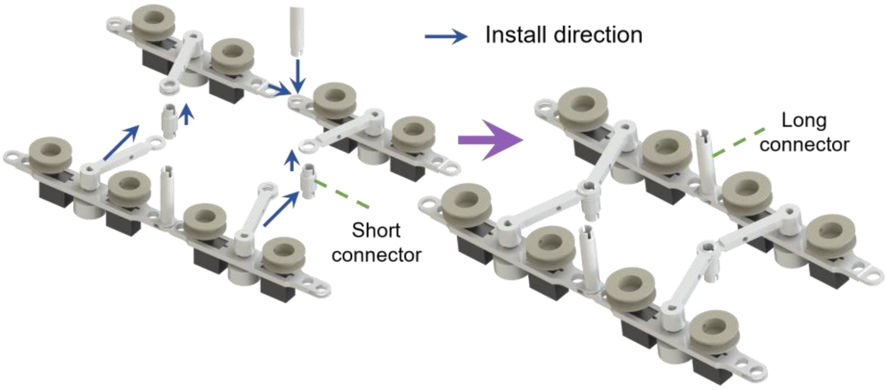

Figure S15. Procedure for assembling EPT unit cell.

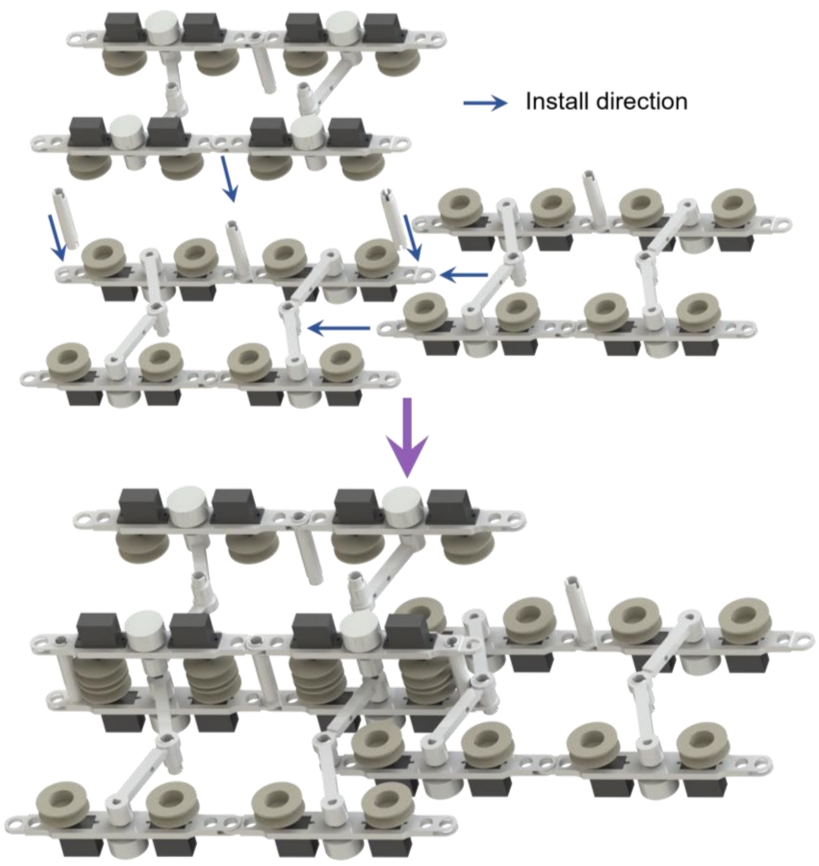

Figure S16. Assembling EPT unit cells.

Movie list

Movie S1.

Principle of elasticity-plasticity transformation. We use the energy method to show the basic principle of EPT material and demonstrate the elasticity-plasticity transformation of a single Lever-strut element.

Movie S2.

From single cell to metamaterial. An EPT metamaterial is built by assembling EPT unit cells. Different responses of an EPT unit in the elastic and plastic modes after stretching and releasing are exhibited.

Movie S3.

Shape sensing of EPT robotic material. We construct the deformation sensing method of the EPT cells and show how to use the isostatic property to reduce the number of adopted sensors.

Movie S4.

Deformation-dependent mechanical property modulation. The EPT material can use its deformation information to control the elastic-plastic transition. The EPT unit cell enters the plastic mode when the cell is stretched laterally into a non-auxetic configuration and staus in elastic mode when the cell is in an auxetic configuration.
